# A Tensile Specimen of Tailor Rolled Blanks with Equal Probability in Yield and Its Mechanical Behavior Analysis

**DOI:** 10.3390/ma11050693

**Published:** 2018-04-28

**Authors:** Sijia Zhang, Xianghua Liu, Lizhong Liu

**Affiliations:** 1 State Key Laboratory of Rolling and Automation, Northeastern University, Shenyang 110819, China; 15840042289@163.com; 2 School of Materials Science and Engineering, Northeastern University, Shenyang 110819, China; liulizhong93@163.com

**Keywords:** tailor rolled blanks (TRB), uniaxial tensile specimen, mechanical behavior, digital image correlation method, finite elements

## Abstract

In this paper, the microstructure and mechanical properties that distribute regulation along the rolling direction of tailor rolled blanks (TRB) were investigated. A tensile specimen with equal probability in yield (EYS) was first designed considering variation both in thickness and in material strength. The uniaxial tension test was carried out with a digital image correlation method to analyze the mechanical behaviors. The results showed that the strain distribution of EYS was homogeneous. From the results, it can be known that a new design philosophy for a TRB tensile specimen is reasonable and EYS is suitable to characterize the mechanical behavior of TRB. The true stress-strain curves of metal in different cross sections of TRB were calculated. On the basis of the true stress-strain curves, a material model of TRB was constructed and then implemented into finite element simulations of TRB uniaxial tensile tests. The strain distribution of numerical and experimental results was similar and the error between the elongation of the specimen after fracture obtained by experiment and FE ranged from 9.51% to 13.06%. Therefore, the simulation results match well with the experimental results and the material model has high accuracy and as well as practicability.

## 1. Introduction

Due to the development of lightweight technology in the automobile industry, tailored blanks based on new material processing technology such as tailor welded blanks (TWB) and tailor rolled blanks (TRB) have been widely studied and well developed recently. Merklein et al. [1] showed the state of the art in scientific research and chances for further scientific investigations concerning tailored blanks. Liu [2] summarized the advantages of TRB, namely, shorter production process, uniform thickness transition, and better surface quality and forming properties compared with TWB and patchwork blanks. Kopp et al. [3] pointed out that the application of TRB is not only limited to the automotive manufacturing industry but can also be found in other technical fields. As a typical lightweight product, the potential of TRB has already been recognized. Despite widespread use, the design and forming process of TRB still presents certain issues. The mechanical properties of TRB are different from those of a constant-thickness blank and the research methods cannot fully draw on the experience of TWB. The main factors influencing the mechanical properties of TRB are the various thicknesses and work-hardening degrees along the rolling direction.

The mechanical behavior of TRB during the forming process has been widely studied in experiments. Kim and Lim [4] investigated the relationship between heat treatment condition and formability of TRB. Furthermore, they proposed an optimized annealing process to increase the formability of TRB of Al-5.5 wt % Mg alloy. Kopp et al. [3] conducted some special deep drawing tests to investigate the forming behavior of TRB. In order to reduce wrinkling during stamp forming, they developed a new type tool with an adaptable blank holder consisting of a multilayer structure to compensate for the thickness difference of TRB. Meyer et al. [5] put the main focus on the further processing of TRB. They concluded that through optimizing the sheet thickness distribution of TRB, the maximum draw depth could be increased and the weight could be reduced. Kleiner et al. [6] successfully manufactured a large-scale component optimized in regard to lightweight construction by utilizing high-pressure sheet metal forming in combination with the use of TRB. Chatti and Kleiner [7] manufactured a front cross member with two thickness transition zones and two crash boxes. The work of Kleiner et al. [6] and Chatti and Kleiner [7] showed the feasibility of the further formation of TRB into complex components in cooperation with reasonable processes.

Experimental investigations of TRB are often combined with numerical studies. Kopp et al. [3] simulated the deep drawing process of TRB in LS-DYNA 3D. The original shell thickness of each node was assigned the corresponding actual thickness of TRB by a special tool. Urban et al. [8] simulated the high-pressure sheet metal forming of TRB, and the results of simulation and experiment agreed well. Lu et al. [9] established a finite element (FE) model which considered variations both in thickness and material strength. With the FE model, the crashworthiness of tailor rolled tubes under different geometric parameters were studied. Numerical simulation enables a better understanding of the mechanical properties and forming behavior during TRB formation.

Accurate modeling of TRB requires knowledge of the mechanical properties of metal in different cross sections. Determination of mechanical properties of TRB is really a difficult task. On the one hand, since the thickness of TRB varies along the rolling direction, the conventional tensile specimen is not appropriate to investigate the mechanical behavior of TRB through uniaxial tensile test. On the other hand, the mechanical properties of TRB are inhomogeneous and cannot be presented by the mechanical properties of metal in any region of the TRB. In order to resolve the aforementioned problem, Zhang et al. [10] proposed, on the basis of uniaxial tensile test data of the thick area and thin area, adopting the Lagrange polynomial interpolation to construct the stress-strain fields of TRB. Shafiei and Dehghani [11] designed a type of tensile specimen with an additional lateral wedge angle. Thus, the cross-sectional area of the specimen is constant. In this way, the mechanical behavior of TRB was studied. However, Zhang et al. [10] did not consider the mechanical properties of transition zone and the interaction between the metal in different regions of TRB during the deformation process. In the tensile specimen designed by Shafiei and Dehghani [11], deformation is controlled by the side with higher load-bearing ability, therefore the strain distribution in this specimen is inhomogeneous.

The purpose of this study is to propose a more reliable method to characterize the complex mechanical behavior of TRB and obtain necessary mechanical properties to model TRB. In this way, the microstructure and mechanical properties that distribute regulation along the rolling direction of TRB were investigated. A new design philosophy for a TRB tensile specimen was proposed based on the mechanical conditions analysis of the specimen under uniaxial tension. A new type of TRB tensile specimen, which differs from the conventional tensile specimen with constant width (CWS) and the tensile specimen with constant cross-sectional area (CAS), was first put forward. The material model of TRB was constructed based on the true stress-strain curves of metal in different cross sections of TRB obtained from uniaxial tensile tests in combination with the DIC method. The material model was then employed in the FE simulations of uniaxial tensile tests and the simulation results were corroborated and verified by experiments.

## 2. Experimental Procedure

### 2.1. Experimental Material

**Figure 1:**
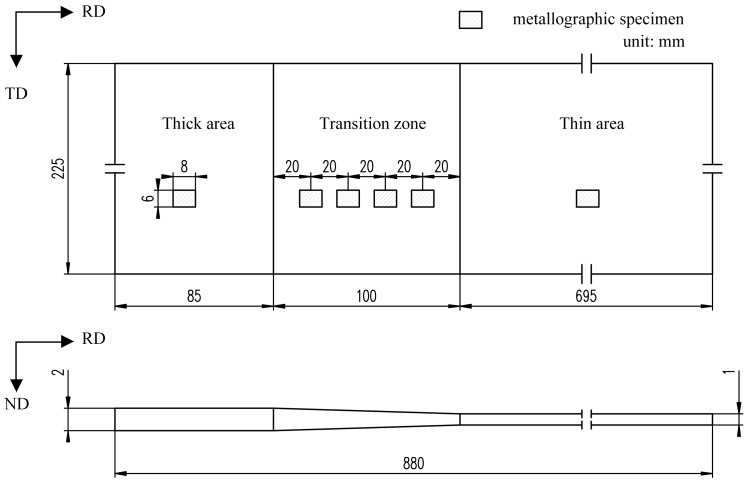
Schematic diagram of the experimental TRB.

The raw material used in the present study was HC340LA steel sheet with the thickness of 2.2 mm, with a chemical composition of C 0.08, Si 0.2, Mn 0.88, P 0.025, S 0.025, Al 0.047 and balanced of Fe (mass fraction, %). The TRB with longitudinal thickness transition zone were produced using a 4-high cold mill by a flexible rolling process. Through controlling the rolling speed and adjusting the roll gap accurately, TRB with target thickness distribution can be obtained. Figure 1 shows the shape and dimensions of the experimental TRB. In addition, the raw material was cold rolled with different thickness reductions of 9%, 18%, 27%, 36%, 45%, and 54%, respectively. Thus, a series of constant-thickness blanks with the thickness of 2.0 mm, 1.8 mm, 1.6 mm, 1.4 mm, 1.2 mm, and 1.0 mm were obtained. After rolling, the TRB and constant-thickness blanks were annealed at 600 °C for 5 h to reduce the effect of work-hardening and inhomogeneity of the mechanical properties.

### 2.2. Experimental Method

In order to investigate the effect of the rolling reduction ratio on the microstructure of HC340LA, metallographic specimens in raw material and in different locations of cold-rolled and annealed TRB were prepared. The location of the metallographic specimens in TRB is shown in Figure 1. The metallographic specimens were mechanically polished and chemically etched with 4% nital. Then, the specimen microstructures in longitudinal section (RD × ND) were observed.

In order to investigate the effect of the rolling reduction ratio on the mechanical properties of HC340LA, uniaxial tensile tests were performed. The tensile specimens of raw material and cold-rolled and annealed constant-thickness blanks were designed according to ASTM E8M. It is noteworthy to mention that, due to the raw material, the deformation path and heat treatment process of the constant-thickness blanks are the same as those of the metal with the corresponding thickness in the TRB. Therefore, the tensile properties of the constant-thickness blanks can be assigned to their corresponding thickness along the TRB of HC340LA.

In order to analyze the mechanical behavior of the TRB tensile specimen and verify the feasibility of the design philosophy for TRB tensile specimen, the uniaxial tensile tests were operated in combination with the digital image correlation (DIC) method. Before the test, the surface of the specimen was painted with black and white colors to form random speckles. During the test, continuous pictures of the deforming specimen were taken by 2 digital cameras to record the whole test process and the frequency of taking photos was 2 pictures per second. Then, the pictures were analyzed by the DIC program to obtain the true strain fields.

All the tensile specimens were stretched under uniaxial force until fracture occurred using the CMT5105 electronic universal testing machine (50 KN) (MTS Systems Corporation, Eden Prairie, MN, USA), and the constant cross-head separation speed was 3 mm/min. For each type of specimen, 3 repeated tests were conducted to validate repeatability.

**Figure 2:**
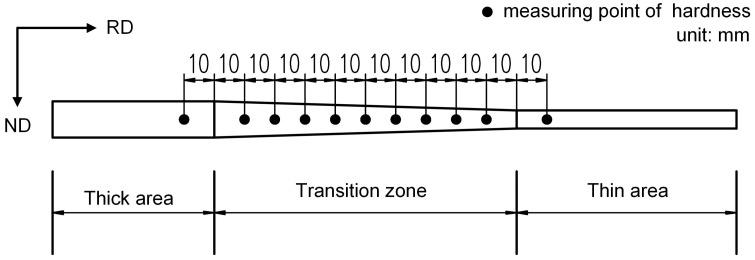
The location of the measuring points of hardness.

Vickers hardness tests were performed to evaluate the effects of the rolling reduction ratio on the mechanical properties of HC340LA as well. The location of the measuring point in TRB is shown in Figure 2. The tests were performed using a KB3000BVRZ-SA universal hardness tester (Leica Microsystems Inc., Wetzlar, Germany). The test force was 196.1 N and the duration of the test was 10 s.

## 3. Design a New Type of Tensile Specimen for TRB

### 3.1. Microstructure and Mechanical Properties Distribution of TRB along Rolling Direction

**Figure 3:**
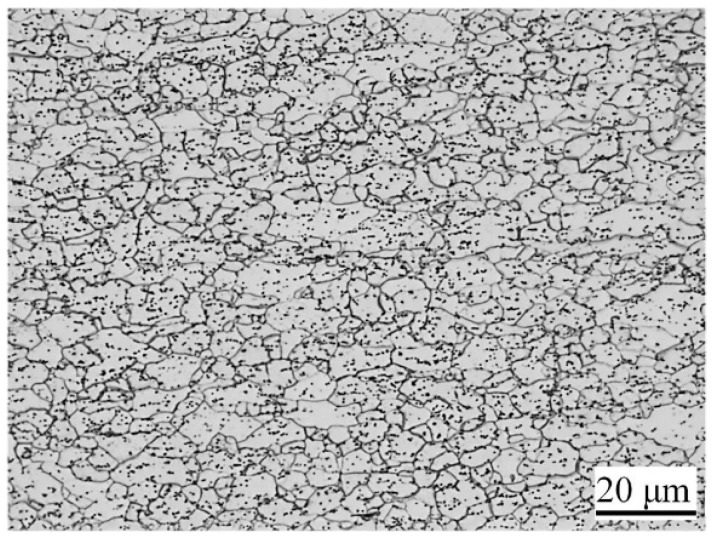
Microstructure of raw material.

Figure 3 shows the microstructure of the raw material. The recrystallized grains with the average size of 9 μm can be observed. The microstructure consists of ferrite and a little pearlite. A considerable amount of granular cementite is distributed along the grain boundary, as well as in grains.

**Figure 4:**
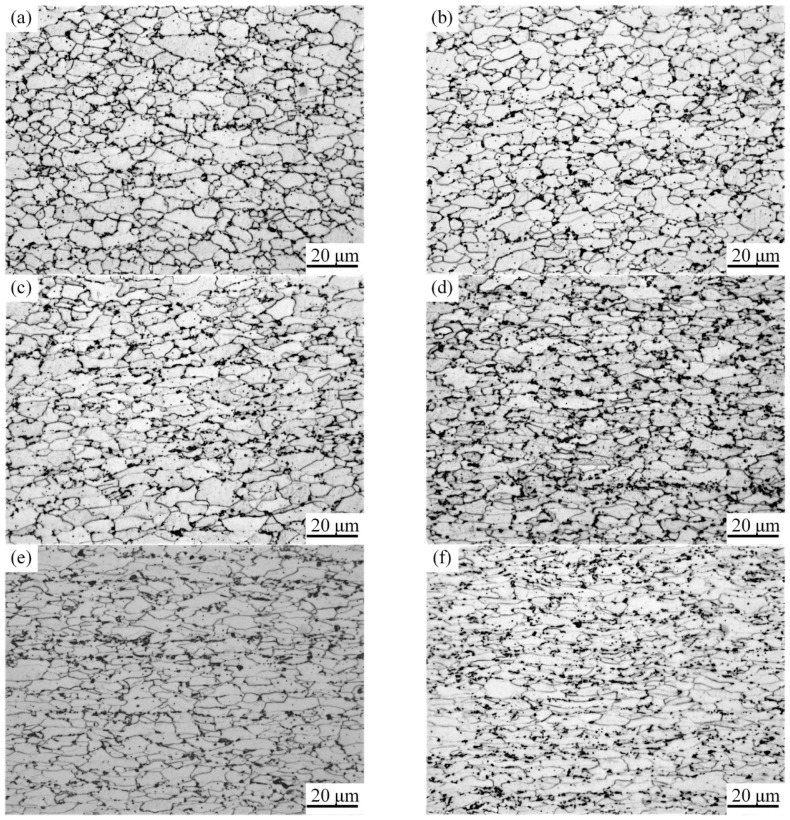
Microstructure of cold-rolled TRB, reduction ratio: (**a**) 9%; (**b**) 18%; (**c**) 27%; (**d**) 36%; (**e**) 45%; (**f**) 54%.

**Figure 5:**
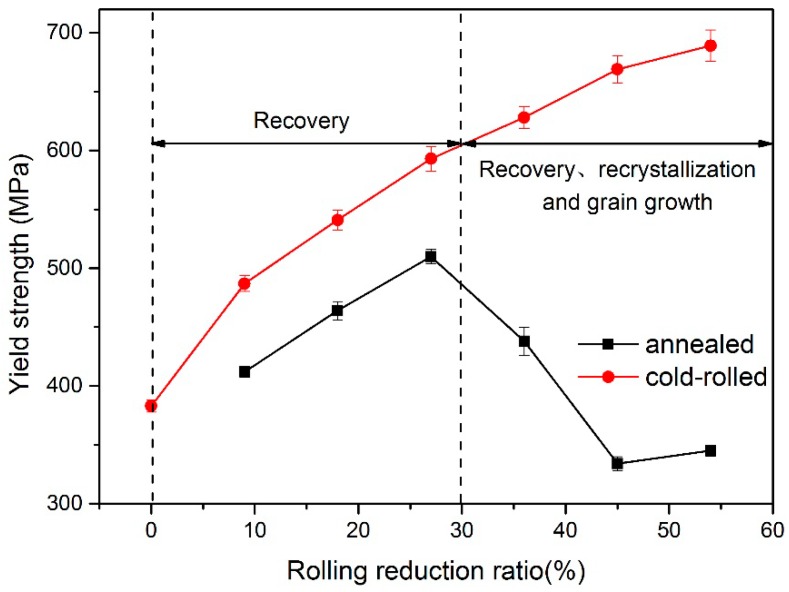
Yield strength of cold rolled and annealed TRB.

Figure 4 and Figure 5 show the microstructure and yield-strength distribution of cold-rolled TRB along the rolling direction, respectively. It can be observed that, with the increase of the rolling reduction ratio, the grains are gradually elongated. Meanwhile, due to the effect of work-hardening, the strength increases approximately in a line.

**Figure 6:**
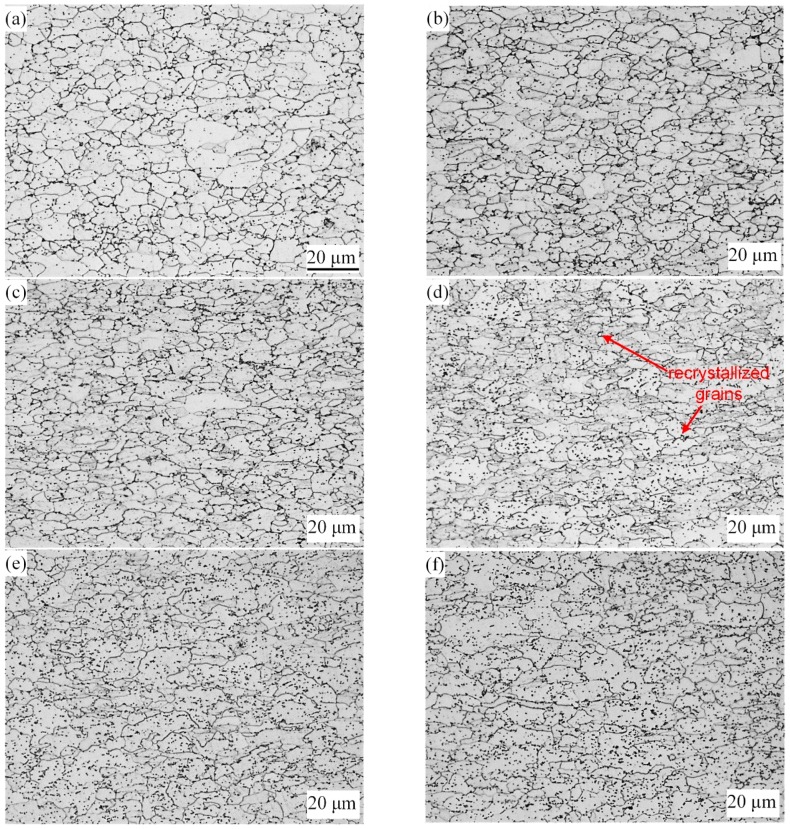
Microstructure of annealed TRB, reduction: (**a**) 9%; (**b**) 18%; (**c**) 27%; (**d**) 36%; (**e**) 45%; (**f**) 54%.

The evolution of microstructures of TRB after annealing is illustrated in Figure 6. From this, we can see that when the rolling reduction ratio is small, i.e., 9%, 18% and 27%, in comparison with the deformed microstructure, no obvious change can be observed in the annealed microstructure. When the rolling reduction ratio is 36%, the microstructure of the specimen after annealing includes flattened grains and a small amount of fine recrystallized grains. In the case of the rolling reduction ratio being more than 45%, the original deformed grains are gradually replaced by equiaxed recrystallized grains. Different reduction ratios can provide different degrees of storage energy for metal which provide the driving force for recovery, recrystallization, and grain growth. The higher the deformation of the metal, the more storage energy in the metal and the lower the temperature needed for recrystallization [12]. In this study, when the rolling reduction ratio was less than a critical value (about 30%), the deformed microstructure of specimen just underwent recovery during the annealing process and the strength decreased slightly. When the rolling reduction ratio was more than the critical value, new recrystallized grains were generated in the original deformed microstructure and the recrystallized grains grew during the annealing process. Furthermore, under the same annealing process, the larger the rolling reduction ratio, the larger the proportion of recrystallized grains in the microstructure. Therefore, under the competition of work-hardening and annealing softening, the strength and hardness of the metal after annealing decreased in different degree.

**Figure 7:**
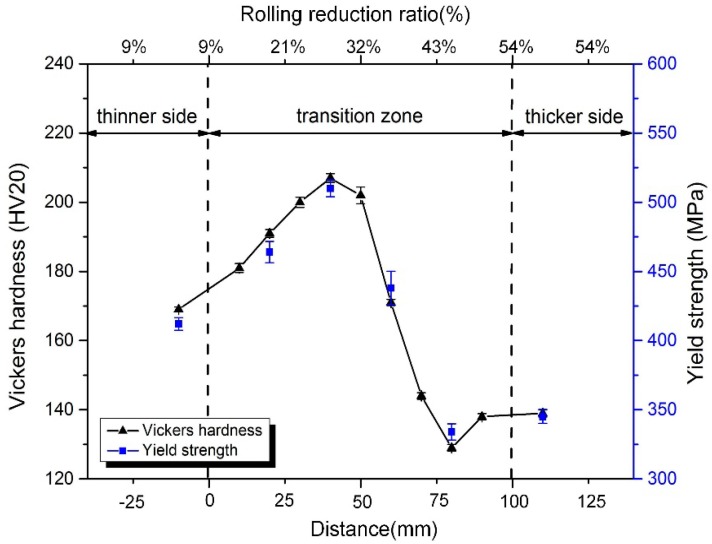
Vickers hardness and yield strength of annealed TRB.

Figure 7 shows the hardness and strength distribution in annealed TRB. As shown in Figure 7, the mechanical properties of TRB along rolling direction is inhomogeneous. The hardness and strength of the metal after annealing increase first and then decrease with the increase of the rolling reduction ratio, reaching the maximum value at the reduction ratio of 27%. The changes of microstructure coincide with the changing regularity of the mechanical properties of the metal after annealing.

### 3.2. Design Philosophy for TRB Tensile Specimen

As stated above, both the thickness and mechanical properties of TRB vary along the rolling direction. In addition, there are no standards for designing TRB tensile specimen. For the traditional method that deals with the standard tensile specimen of a constant-thickness blank to obtain the mechanical properties of each part of TRB, there are some disadvantages, such as: (1) it is an indirect method that will cause deviation; (2) the process is complex and the workload is heavy; and (3) for some researchers and customers, there are insufficient conditions to prepare the constant-thickness blank. Therefore, it is necessary to propose a design philosophy for a TRB tensile specimen to characterize the mechanical behavior and obtain the mechanical properties of each part of TRB.

**Figure 8:**
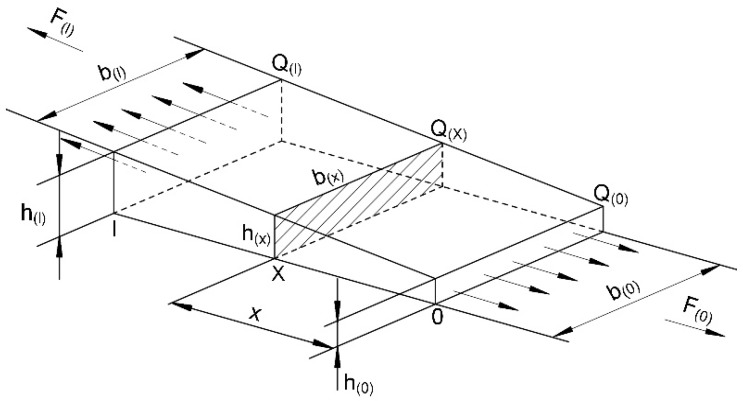
Schematic of position of cross section *X* and force conditions of TRB under uniaxial tension.

During the uniaxial tensile test for TRB, it is assumed that the stress acting on a cross section of the specimen is uniformly distributed. Figure 8 shows the position of cross section X and force conditions of TRB during a tensile test. According to the force balance during the uniaxial tensile test, as shown in Equation (1), the tensile force on each cross section of the specimen is equal:

(1) $$Q_{\left(0\right)}\times A_{\left(0\right)}=Q_{\left(x\right)}\times A_{\left(x\right)}=Q_{\left(l\right)}\times A_{\left(l\right)}=F_{\left(0\right)}=F_{\left(x\right)}=F_{\left(l\right)}=F\;0\leq x\leq l$$

where *F* is the tensile force provided by the tensile testing machine. *A*(*x*) is the area of cross section *X*. *Q*(*x*) and *F*(*x*) are uniformly distributed loads and forces acting on the cross section *X*, respectively.

The yield strength of metal in cross section *X* of TRB is *σ*(*x*), and the thickness and width of this cross section are *h*(*x*) and *b*(*x*), respectively. When the metal enters into the yield stage under the uniaxial tensile force and plastic deformation starts to occur, the stress can be express by Equation (2):

(2) $$\sigma_{\left(x\right)}=\frac{F}{b_{\left(x\right)}\times h_{\left(x\right)}}$$

Assuming the mechanical properties of metal in the same cross section are equal, on the basis of Equations (1) and (2), the width of cross section *X* can be determined by Equation (3):

(3) $$b_{\left(x\right)}=\frac{F}{\sigma_{\left(x\right)}\times h_{\left(x\right)}}=\frac{\sigma_{\left(l\right)}\times h_{\left(l\right)}\times b_{\left(l\right)}}{\sigma_{\left(x\right)}\times h_{\left(x\right)}}$$

According to Equation (3), the width of each cross section of the TRB tensile specimen is calculated. Thus, the shape and dimensions of the TRB specimen can be obtained. However, because the thickness of TRB varies along the rolling direction, the yield strength of metal in the location of the transition zone cannot be obtained by a uniaxial tensile test like with blanks of a constant thickness.

Strength is a mechanical property parameter that is directly proportional to hardness [12,13,14,15,16,17], i.e., the strength and hardness of TRB have a similar distributed regulation (Figure 7). In addition, the hardness of any location in the TRB can be measured directly using a macro hardness tester. Therefore, Equation (3) can be rewritten to Equation (4):

(4) $$b_{\left(x\right)}=\frac{HV_{\left(l\right)}\times h_{\left(l\right)}\times b_{\left(l\right)}}{HV_{\left(x\right)}\times h_{\left(x\right)}}$$

In Equation (4), *HV*_(*x*)_ is the hardness of cross section *X*, and *HV*_(*l*)_ is the hardness of cross section *l*.

It is noteworthy to point out that even though the thickness and mechanical properties are numerous, each cross section of the specimen with the width calculated according to Equation (4) can enter into the yield stage at the same time. However, it is difficult to guarantee each cross section of the specimen will yield synchronously in practice for the statistics viewpoint. Therefore, a more rigorous expression should be that the probability of each cross section of the specimen entering into the yield stage is equal during the tensile process. Furthermore, we propose a design philosophy for a TRB tensile specimen, namely, assuming that the mechanical properties of metal in the same cross section of TRB are equal, then the shape and dimensions of the tensile specimen should make the metal in every cross section of the specimen have the same probability to start to yield. For the sake of simplicity, this design philosophy is called “equal probability in yield philosophy” and Equation (4) is the mathematical expression of this.

When utilizing the above philosophy to design the TRB tensile specimen, it is necessary to select some special cross sections at first. Then, the hardness of each cross section should be tested. After, the width of each special cross section can be calculated according to Equation (4). Furthermore, the shape and dimensions of the TRB specimen can be determined by interpolation.

### 3.3. Specimen Design and Preparation

On the basis of the dimensions of the experimental TRB (Figure 1) and the results of the hardness test (Figure 7), utilizing the aforementioned design philosophy, a new type of uniaxial tensile specimen, i.e., EYS, was designed in this study. Furthermore, another 2 types of specimens, that is, a conventional tensile specimen with constant width (CWS) and a tensile specimen with constant cross-sectional area (CAS) were machined as objects for comparison. The axial direction of the specimen was the rolling direction. The design method refers to the standard ASTM E8M.

**Figure 9:**
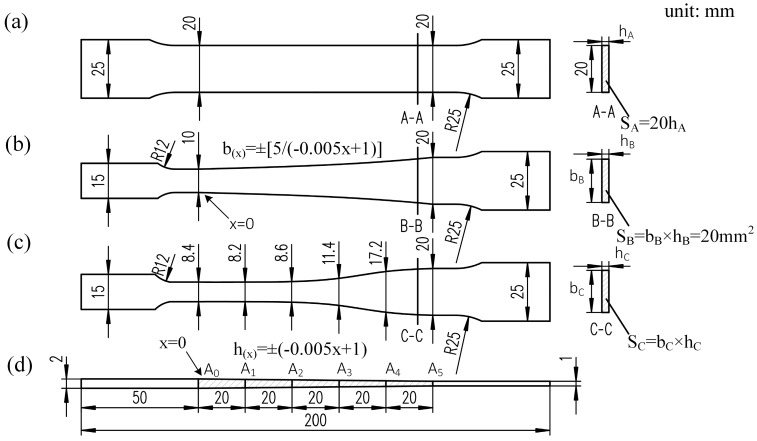
Dimensions of TRB tensile specimens (**a**) CWS; (**b**) CAS; (**c**) EYS; (**d**) side view.

The schematic diagram of 3 types of tensile specimens is shown in Figure 9. For convenience to compare, the overall lengths of the specimens were all set to 200 mm and the widths of the cross sections with the smallest thickness were all set to 20 mm.

**Figure 10:**
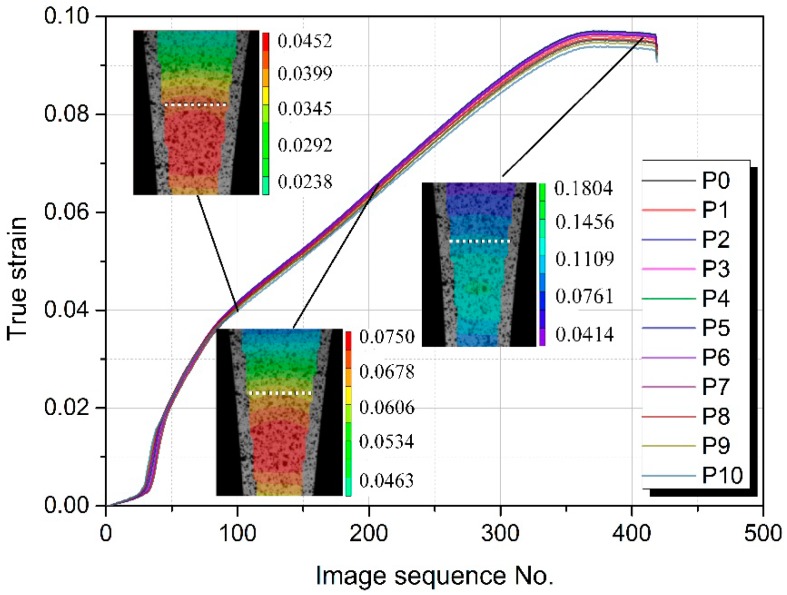
Length strain of each node during the test based on the DIC results.

The sides of the conventional specimen were 2 parallel lines, while the sides of CAS and EYS were 2 symmetric spline curves. The modified geometry makes the specimen, when under a load, no longer in uniformly distributed pure axial tension. In order to ensure the influence of the modified geometry on the strain distribution on the cross section, 11 nodes (from P0 to P10) on a perpendicular line through the thickness transition zone were selected and the major strain vs. image sequence number curves of selected nodes were constructed as shown in Figure 10. It can be seen from Figure 10, preliminarily, that at the same time, the true strain of each node is almost equal. To quantify the uniformity of the strain distribution further, the coefficient of variation of the major strains was utilized according to Equation (5):

(5) $$C.V=\frac{\sqrt{\frac{1}{n}\sum_{i=1}^{n}{\left(\varepsilon_{i}-\varepsilon_{a}\right)}^{2}}}{\varepsilon_{a}}$$

where *C.V* is the variation index of strain distribution, *ε*_i_ is the length strain of each node, *ε_a_* is the average value of the length strain of each node, and *n* is the number of the nodes.

Taking EYS as the research object, the *C.V* of length strains at the moments of 50, 100, and 202 s are 1.24%, 2.10%, and 3.46%, respectively by calculating. As the *C.V* increases steadily until the occurrence of fracture, the *C.V* of the last image is less than 5%. Therefore, we can conclude that the strains on the cross section of the specimen are uniform during the test and the effect of the modified geometry on the strain distribution can be negligible.

## 4. Results and Discussion

### 4.1. Experimental Results and Discussion

#### 4.1.1. Observation of TRB Specimens

**Figure 11:**
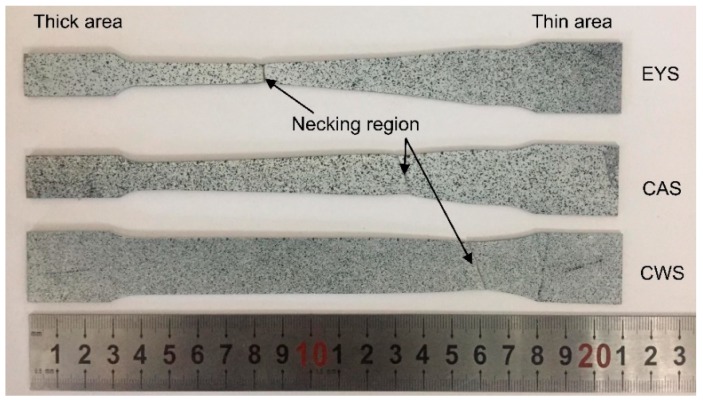
TRB specimens after tensile test.

TRB specimens with necking by tensile tests are shown in Figure 11. It can be observed that the necking region occurs in the thin area of the CWS, whereas it happens on the transition zone of CAS and EYS. Moreover, in EYS, the fracture position is closer to the middle of the specimen. Table 1 shows the elongation values of reduced sections of TRB specimens and those of transition zones of TRB specimens. As shown in this table, there is little difference in the elongation of reduced sections. The elongation of the transition zone of EYS is 11%, which is 124% and 39% higher than that of CWS (4.9%) and CAS (7.9%), respectively. These results indicate that the homogeneity of elongation in EYS is better than that in CWS and CAS.

#### 4.1.2. Strain Distribution of TRB Specimens

**Figure 12:**
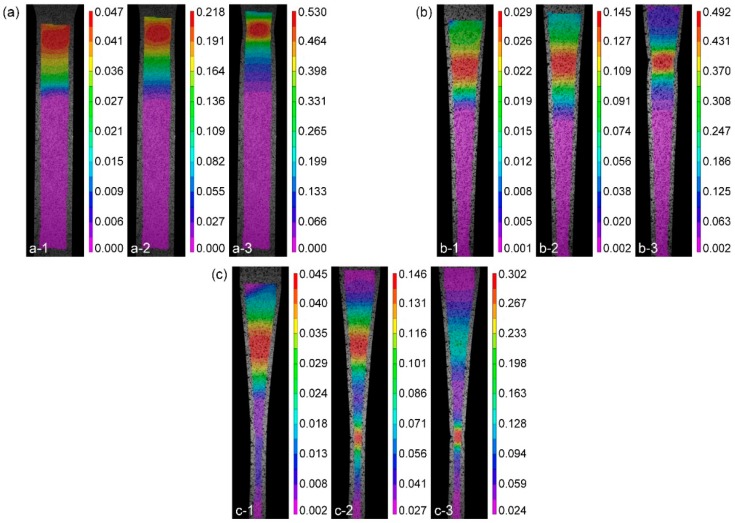
Length strain distribution of the specimen during the test: (**a**) CWS; (**b**) CAS; (**c**) EYS.

The length strain distribution of the TRB specimens is shown in Figure 12. In Figure 12a, the strain of CWS is not uniformly distributed. The plastic deformation occurs mainly in the thin area and the region with a thickness of 1.0–1.2 mm in the transition zone. The strain localization starts at an early stage and the inhomogeneous degree of strain distribution increases during the deformation process. The difference between the maximum strain and minimum strain before fracture is 0.53. Similar phenomenon is also observed in CAS as shown in Figure 12b. In comparison with CWS, the inhomogeneous degree of strain distribution decreases slightly. The plastic deformation mainly occurs in the region with a thickness of 1.0–1.4 mm in the transition zone. The difference between the maximum strain and minimum strain before fracture is reduced to 0.49. As shown in Figure 12c, during the tensile test, the strain distribution of EYS is relatively homogeneous. At the early stage of the tensile test, the strain of the metal in the cross section with a thickness of 1.3 mm is the largest (Figure 12(c-1)). With the development of the deformation process, the strain peak shifts to the metal near the thickness of 1.65 mm (Figure 12(c-3)). Plastic deformation occurred in the whole reduced section when the specimen fractured. The difference between the maximum strain and minimum strain before fracture is 0.275.

**Figure 13:**
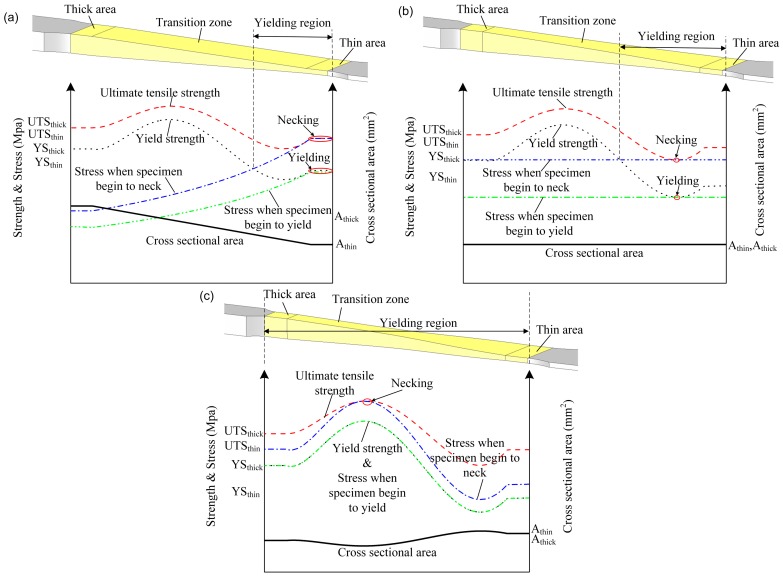
Stress distribution of the specimen during the test: (**a**) CWS; (**b**) CAS; (**c**) EYS.

The stress distribution on the cross section of the specimen during the test shown in Figure 13 can be utilized to explain the above observation. According to Equation (2), the stress on the cross section changes inversely with the area of the cross section. As shown in Figure 13a, during the deformation process, the stress on the cross section of the thin area reaches the yield strength first and the specimen begins to yield. With the progression of the test, necking occurs in the thin area, and at the same time, the stresses on some cross sections are still less than the yield strength and no plastic deformation occurs on these cross sections. Therefore, the strain distribution is highly inhomogeneous and the plastic deformation just occurs in a small region. In Figure 13b, for CAS, the stress on each cross section is equal. Due to the variation in material strength of TRB, under the same stress, the specimen will yield first in a location where yield strength is the lowest. This is due to the yield strengths of some cross sections being larger than the ultimate tensile strength of the cross section where the necking occurs. Most regions of the specimen do not enter into the yield stage and only elastic deformation occurs there. This is because EYS is designed according to the equal probability in yield philosophy, which considers variation both in thickness and material strength. As shown in Figure 13c, in theory, the whole reduced section will begin to yield at the same time. Therefore, plastic deformation can occur in the whole reduced section.

Based on the above results, since EYS is able to eliminate the influence of various thickness and mechanical properties on strain distribution of the specimen, the strain distribution of EYS is relatively homogeneous. The elongation of the transition zone of EYS is higher than that of CWS and CAS, which means that the plastic deformation in EYS is more sufficient. For the three types of specimens investigated in this study, EYS is suitable for obtaining the stress-strain relationships of metal in different cross sections and characterizing the mechanical behavior of TRB.

#### 4.1.3. Force-Displacement Curves of Specimens

Zhao et al. [18] pointed out that during the uniaxial tensile test, the precondition of the iso-strain assumption and the iso-stress assumption itself should be achieved at the same time. Otherwise, the stress-strain curve of the specimen obtained by the uniaxial tensile test does not have scientific significance. Due to the variation both in thickness and in material strength along the rolling direction of TRB, the force-displacement curve of TRB specimens was plotted in this study.

**Figure 14:**
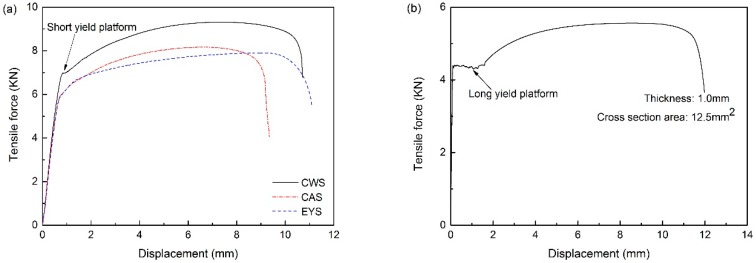
Force-displacement curves: (**a**) TRB specimens and (**b**) specimen of blank with constant thickness.

Figure 14 shows the force-displacement curves of different TRB specimens and the specimen cut out from the thin area of the experimental TRB. According to the dimensions of TRB tensile specimens and the yield strengths of constant-thickness blanks tested before, for the three types of specimens (CWS, CAS, and EYS), the tensile forces required to yield should be 6.90, 6.68, and 6.90 KN, respectively. This disagrees with Figure 14a. It means that the metal with the lowest yield strength in the experimental TRB is not located on the selected special cross sections. Taking the force-displacement curve of CAS as the research object, the tensile force required to yield is about 5.6 KN and the lowest yield strength of the metal is calculated as 280 MPa. In combination with the strain distribution in Figure 12b, it can be seen that the specimen first yields at the thickness of 1.23 mm, i.e., the metal with the lowest yield strength of 280 MPa is located at the position with a thickness of 1.23 mm in the TRB.

In Figure 14b, it is observed that there is a long yield plateau in the force-displacement curve. However, there is no similar phenomenon in Figure 14a—only a short yield plateau in the force-displacement curve of CWS. This phenomenon is due to various mechanical properties along the rolling direction of TRB. As the mechanical properties are inhomogeneous, the yield plateaus of metal in different regions of TRB are all very short and will appear in different stages during the tensile test. For CWS, as the deformation mainly occurs in the thin area and the thin area in the specimen is short, a short yield plateau can be observed. For CAS and EYS, the deformation mainly occurs in the thickness transition zone, so the tensile force keeps rising and the long yield plateau cannot be observed in the force-displacement curve.

#### 4.1.4. The True Stress-Strain Curve of Metal in TRB

During the tensile test, the true length strain *ε_l_* and true width strain *ε_b_* of nodes on the surface of a specimen can be measured directly through the two-dimensional digital image correlation program. According to the law of constancy of volume, the true thickness strain *ε_a_* shall be calculated by using the equation: *ε_a_* = − (*ε_l_* + *ε_b_*). The tensile force F on a specimen in the experiment can be obtained through the electronic tensile testing machine. For the metal in a cross section of TRB, since *ε_a_*, *ε_b_*, *ε_l_*, and *F* are determined, the true stress *σ* during the test can thus be calculated and the true stress-strain curve can be constructed according to the following steps:

1. Measure the original width *b*_0_ and the original thickness *h*_0_ of the cross section;
2. The width *b_t_* (*t* = 0, 1, 2 s, …) and thickness *h_t_* of the cross section at time *t* can be calculated by
  (6) $$b_{t}=b_{0}e^{\varepsilon_{b}}$$
  (7) $$h_{t}=h_{0}e^{\varepsilon_{a}}$$

The area *A_t_* is

(8) $$A_{t}=b_{t}h_{t}=b_{0}h_{0}e^{\left(\varepsilon_{b}+\varepsilon_{a}\right)}=b_{0}h_{0}e^{-\varepsilon_{l}}$$

Furthermore, the true stress *σ_t_* of metal in the cross section can be calculated by Equation (9)

(9) $$\sigma_{t}=\frac{F_{t}}{A_{t}}=\frac{F_{t}}{b_{0}h_{0}e^{-\varepsilon_{l}}}=\frac{F_{t}}{b_{0}h_{0}}e^{\varepsilon_{l}}$$

According to the measured true strain *ε_l_* and corresponding true stress *σ_t_* at different moments, the true stress-strain curve of metal in the selected cross section is plotted.

**Figure 15:**
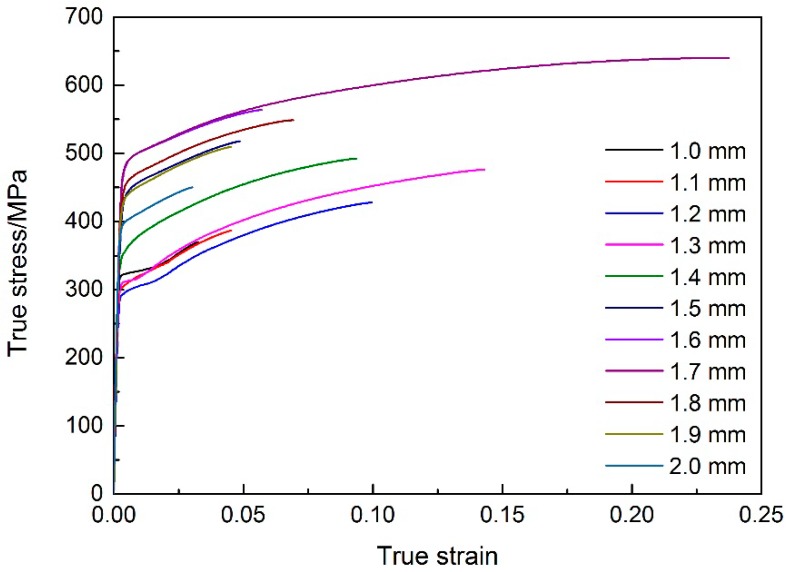
True stress-strain curves of materials in experimental TRB.

In this work, the true stress-strain curves of metal in different cross sections with thicknesses of 1.0, 1.1, 1.2, 1.3, 1.4, 1.5, 1.6, 1.7, 1.8, 1.9, and 2.0 mm in the transition zone of EYS were calculated and are shown in Figure 15. As mentioned above, the flexible rolling process and specific annealing process lead to inhomogeneous microstructures in TRB. Therefore, the stress-strain relationship in different thicknesses of TRB is different. No yield plateaus have been found in the true stress-strain curves. The yield strength of metal in TRB is lower than that in constant-thickness blanks with the same thickness tested before. Two main reasons for this phenomenon are: (1) there is an interaction between the metal in different regions of TRB during the test, and the coordinated deformation occurs in the specimen, which has an impact on the mechanical behavior of each metal; (2) true strain can be measured with higher accuracy by the DIC method.

The true stress-strain curves calculated in this study are incomplete due to insufficient tension deformation of metal in the tensile specimen except in the fracture location. In order to characterize the mechanical behavior of experimental TRB as accurately as possible, the incomplete true stress-strain curves were fitted to the Hollomon equation:

(10) $$\sigma =\{\begin{array}{cc} E\varepsilon & \left(\sigma \leq \sigma_{y}\right)\\ K\varepsilon^{n} & \left(\sigma >\sigma_{y}\right) \end{array}$$

where *E* is the Young’s modulus, *K* is strength coefficient, *n* is tensile strain hardening exponent, and *σ_y_* is yield strength. The material parameters of the fitted curves are shown in Table 2.

### 4.2. Numerical Analysis and Discussion

#### 4.2.1. Finite Element Model

**Figure 16:**
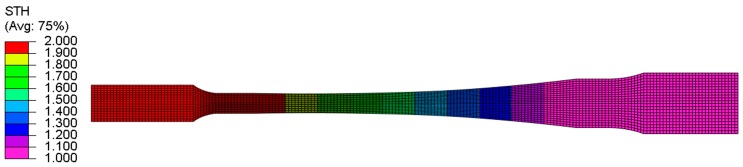
Geometric model of EYS.

The uniaxial tensile simulations on different specimens of experimental TRB were performed by using a commercially available finite element code ABAQUS 6.14. Because of the tensile test is a quasi-static process, the ABAQUS/Explicit was used to carry out the simulations. The shell elements (S4R) were adopted for simulation and the average element edge-length is 1 mm. In the thickness direction, 9 integration points were selected to improve the simulation accuracy. The geometric parameters of the model are same as those of the specimen. The nodes of the grip section with the thickness of 2 mm were fixed. The nodes of the grip section with the thickness of 1 mm were tied to a reference point, then a speed of 3 mm/min was assigned to the reference point. Figure 16 shows the geometric model of EYS, and the number of elements is 4242. It is noteworthy to mention that, through the method of predefining a thickness field, continuously various thicknesses were assigned to the specimen. Because the anisotropy in the material is not considered, the Mises yielding criterion was used in the computational model.

**Figure 17:**
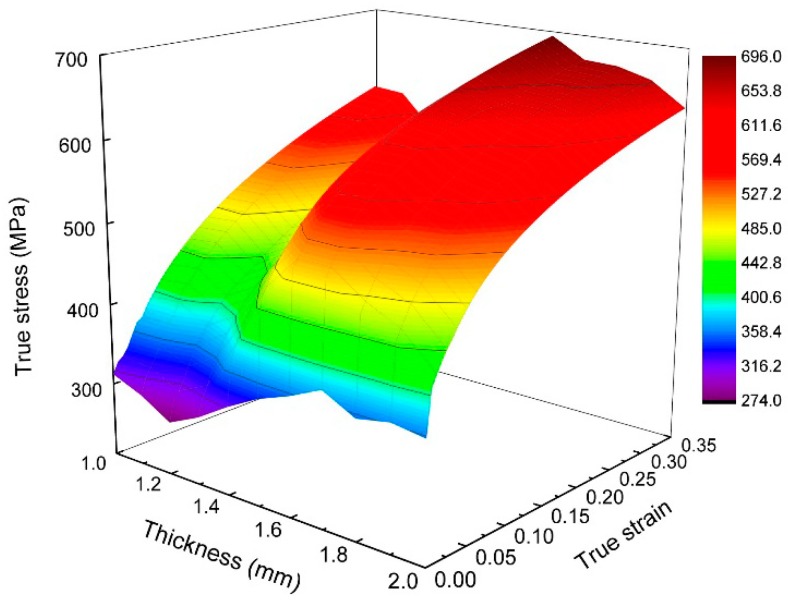
Material model of TRB.

For TRB simulation, one of the most important parts is selecting an appropriate material model. In this study, the Young’s modulus and Poisson’s ratio were set to 206 GPa and 0.3, respectively, and the stress value, which produces 0.2% residual deformation, was taken as yield strength. Then, on the basis of the true stress-strain curves obtained from tensile tests, the material model of TRB as shown in Figure 17 was constructed by the method of interpolation, implementing the material model into simulations of TRB tensile tests. The mechanical parameters obtained from the model were assigned to the element of the corresponding thickness.

#### 4.2.2. Simulation Results and Comparison

**Figure 18:**
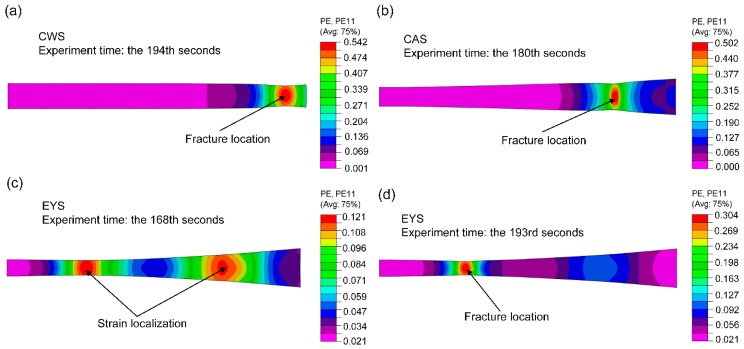
Strain distribution under different conditions of FE results. (**a**) specimen: CWS, time: 194 s; (**b**) specimen: CAS, time: 180 s; (**c**) specimen: EYS, time: 168 s; (**d**) specimen: EYS, time: 193 s.

The FE simulations of uniaxial tensile tests were performed. Figure 18 shows the length strain distribution under different conditions of simulation results. In comparison with the length strain fields obtained by the DIC method shown in Figure 12(a-3), (b-3), (c-2), and (c-3), respectively, consistent strain distribution of numerical and experimental results can be observed. In all the cases, the FE predictions of the maximum strain match well with the experimental values. The fracture locations and strain localization are also predicted accurately in the FE simulations. In Figure 18c, two strain peaks are observed and in Figure 18d, the number of strain peaks reduces to only one. It means that the phenomenon of strain peak transfer in the experimental results are also reflected in FE result.

**Figure 19:**
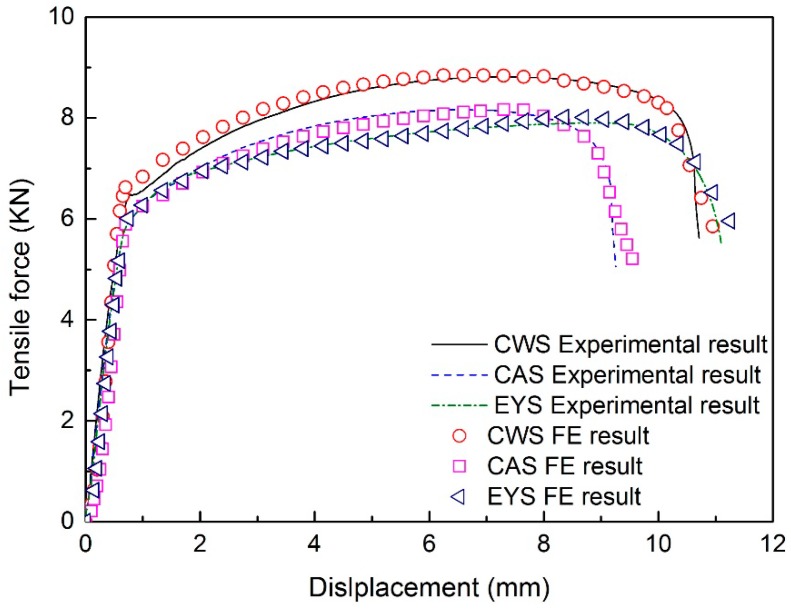
Force-displacement curves of experimental and FE results.

Based on the simulations of tensile tests, the force-displacement curves are determined and shown in Figure 19. It can be seen that the predicted force-displacement curves agree with the experimental results. In addition, there are no long yield plateaus based on the simulation, which is in accordance with the experimental results. Thus, the applicability of the material model of TRB constructed on the basis of EYS and the DIC method is verified.

Nevertheless, the simulation underestimates the overall length of the specimen after fracture (Table 3) and there is a small difference in the necking stage of the force-displacement curves between the experimental and FE results. It could be due to the fact that the mechanical properties of TRB are continuously varying along the rolling direction, while in this study, the material model is constructed by the interpolation method on the basis of 11 true stress-strain curves. There is still a slight difference between the material model and real mechanical properties of TRB. In order to model the deformation process of TRB as accurately as possible, more true stress-strain curves of metal in different cross sections of TRB should be calculated. In addition, the true stress-strain curve obtained by the DIC method is incomplete and the rest of the curve is obtained by nonlinear fitting. This might also contribute to the error between the experimental and FE results.

## 5. Conclusions

In this study, the effects of the rolling reduction ratio on the microstructure and mechanical properties of TRB were investigated and the distributed regulation of microstructure and mechanical properties along the rolling direction of TRB was obtained. Then, a new design philosophy for TRB tensile specimen, i.e., the equal probability in yield philosophy, was proposed. Furthermore, a new type of tensile specimen, namely EYS, was designed. Another two types of tensile specimens, CWS and CAS, were machined for comparison purposes. The mechanical behaviors of these three types of TRB tensile specimens were compared and analyzed by experiment and the experimental results were verified through numerical modeling. The following concluding remarks can be drawn accordingly:

1. Under the combined influence of work-hardening and annealing softening, the mechanical properties of TRB vary along the rolling direction. The hardness and strength of annealed TRB increase first and then decrease with the increase of the rolling reduction ratio, reaching the maximum value at a reduction ratio of 27%;
2. The variation index of the major strain distributions on the cross section of EYS ranges from 1.24% to 3.46%, which is lower than 5%. This indicates that the strains on the cross section of the specimen are uniform during the test and the effect of modified geometry on the strain distribution can be negligible;
3. After tensile tests, the elongation of a reduced section of EYS is 9.3%, which is 5% and 19% higher than that of CWS (8.9%) and CAS (7.8%), respectively. In addition, the elongation of the transition zone of EYS is 11%, which is 124% and 39% higher than that of CWS (4.9%) and CAS (7.9%). The plastic deformation of EYS is more sufficient;
4. The length strain fields of specimens during the deformation process were measured by the DIC method. It is noted that the difference between the maximum and minimum strains in EYS before fracture is 0.275, while that in CWS and CAS are 0.53 and 0.49, respectively. The strain distribution of EYS is more homogeneous compared with the other two types of specimens;
5. There is a long yield plateau in the force-displacement curve of the constant-thickness blank, while a short yield plateau can be observed in the force-displacement curve of CWS and no yield plateau can be observed in the force-displacement curve of CAS and EYS;
6. On the basis of data obtained by the DIC method in a uniaxial tensile test, the true stress-strain curves of metal in 11 different cross sections of TRB were calculated. A material model of TRB was constructed and then implemented into the FE simulations of uniaxial tensile tests;
7. The strain distribution of numerical and experimental results are similar and the fracture locations and strain localization are predicted accurately in the FE simulations. The error between the elongation of the specimen after fracture obtained by experiment and FE ranges from 9.51% to 13.06%. It can be concluded that the FE results are in good agreement with the experimental results. Thus, the applicability of the material model was verified.

In summary, the new design philosophy for a TRB tensile specimen is scientific and reasonable. EYS is suitable to characterize the mechanical behavior of TRB. Combining the DIC method and EYS, the true stress-strain curves of metal in any region of TRB can be calculated with high accuracy, which is of great significance to establish a finite element model for TRB.

## Figures and Tables

**Table 1:** Elongation of reduced sections and transition zones of TRB specimens.

Specimen	Elongation of Reduced Section (%)	Elongation of Transition Zone (%)
CWS	8.9	4.9
CAS	7.8	7.9
EYS	9.3	11.0

**Table 2:** Material parameters of the fitted curves.

Thickness (mm)	Strength Coefficient: *K* (MPa)	Tensile Strain Hardening Exponent: *n*
1.0	742.00	0.21
1.1	731.00	0.20
1.2	679.14	0.20
1.3	703.18	0.19
1.4	747.52	0.17
1.5	790.23	0.14
1.6	770.65	0.11
1.7	755.32	0.11
1.8	758.00	0.12
1.9	745.35	0.12
2.0	727.00	0.14

**Table 3:** Elongation of the specimens after fracture of experimental and FE results.

Specimen	Elongation of the Specimens after Fracture (mm)		Error (%)
	Experiments	Simulations	
CWS	10.72	9.70	−9.51
CAS	9.26	9.00	−2.81
EYS	11.10	9.65	−13.06

Note: Errors were computed as $\frac{L_{\exp eriments}-L_{simulations}}{L_{\exp eriments}}\times 100\%$, where *L* represent a variable.
